# CSF evidence of pericyte damage in Alzheimer’s disease is associated with markers of blood-brain barrier dysfunction and disease pathology

**DOI:** 10.1186/s13195-019-0534-8

**Published:** 2019-09-14

**Authors:** J. S. Miners, P. G. Kehoe, S. Love, H. Zetterberg, K. Blennow

**Affiliations:** 1 Dementia Research Group, Clinical Neurosciences, Bristol Medical School, University of Bristol, Level 1, Learning and Research Building, Southmead Hospital, Bristol, BS10 5NB UK; 2 Department of Psychiatry and Neurochemistry, Institute of Neuroscience and Physiology, the Sahlgrenska Academy at the University of Gothenburg, S-431 80 Mölndal, Sweden; 3 Clinical Neurochemistry Laboratory, Sahlgrenska University Hospital, S-431 80 Mölndal, Sweden; 4 Department of Neurodegenerative Disease, UCL Institute of Neurology, Queen Square, London, WC1N 3BG UK; 5 UK Dementia Research Institute at UCL, London, WC1E 6BT UK

**Keywords:** Platelet-derived growth factor receptor β, PDGFRβ, Cerebrospinal fluid, CSF, CSF albumin, Alzheimer’s disease

## Abstract

**Background:**

We aimed to assess the relationship between levels of a cerebrospinal fluid (CSF) marker of pericyte damage, soluble platelet-derived growth factor receptor β (sPDGFRβ) and CSF markers of blood-brain barrier (BBB) integrity (CSF albumin and CSF/serum albumin ratio) and disease pathology (reduced CSF Aβ42 and elevated CSF total and phosphorylated tau) in Alzheimer’s disease (AD).

**Methods:**

sPDGFRβ and albumin were measured by sandwich ELISA in ante-mortem CSF from 39 AD and 39 age-matched controls that were grouped according to their biomarker profile (i.e. AD cases t-tau > 400 pg/mL, p-tau > 60 pg/mL and Aβ42 < 550 pg/mL). sPDGFRβ was also measured in matched serum and CSF samples (*n* = 23) in a separate neurologically normal group for which the CSF/serum albumin ratio had been determined.

**Results:**

CSF sPDGFRβ level was significantly increased in AD (*p* = 0.0038) and correlated positively with albumin (*r* = 0.45, *p* = 0.007), total tau (*r* = 0.50, *p* = 0.0017) and phosphorylated tau (*r* = 0.41, *p* = 0.013) in AD but not in controls. CSF sPDGFRβ did not correlate with Aβ42. Serum and CSF sPDGFRβ were positively correlated (*r* = 0.547, *p* = 0.0085) in the independent neurologically normal CSF/serum matched samples.

**Conclusions:**

We provide further evidence of an association between pericyte injury and BBB breakdown in AD and novel evidence that a CSF marker of pericyte injury is related to the severity of AD pathology.

**Electronic supplementary material:**

The online version of this article (10.1186/s13195-019-0534-8) contains supplementary material, which is available to authorized users.

## Background

Alzheimer’s disease (AD) and vascular dementia (VaD) together account for most cases of dementia. Cerebral hypoperfusion, neurovascular uncoupling and blood-brain barrier (BBB) breakdown are defining features of VaD, but there is now compelling evidence they are also major contributors to cognitive decline and disease pathology in the early stages of AD (reviewed in [1, 2]). Pericytes are a heterogeneous population of mural cells that are highly enriched within the brain where they regulate blood flow and maintain vascular homeostasis. Histological and biochemical assessment of post-mortem brain tissue has revealed significant pericyte loss in AD, associated with a reduction in the pericyte marker platelet-derived growth factor receptor β (PDGFRβ) [3, 4].

PDGFRβ is shed from human pericytes (but not vascular smooth muscle cells) when they are cultured under hypoxic conditions (1% oxygen for 48 h) or exposed to Aβ peptides [5]. Cell membrane shedding of PDGFRβ was shown to be mediated by ADAM10 cleavage [6]. Elevation of the level of soluble PDGFRβ (sPDGFRβ) in the CSF was associated with evidence on neuroimaging of BBB leakiness within the hippocampus in normal ageing and in mild cognitive impairment [7]. Membrane shedding of PDGFRβ from injured pericytes explains the inverse relation between the loss of pericytes and reduction in PDGFRβ in the brain tissue and the elevation of sPDGFRβ in the CSF in AD.

Nation et al. [6] reported increased CSF sPDGFRβ and regional BBB leakiness in pre-clinical AD and reported that CSF sPDGFRβ predicted cognitive decline independently of CSF Aβ or tau level. The findings are in keeping with data from PDGFRβ-deficient mice [8], indicating that pericyte loss and BBB leakiness contribute to cognitive decline and AD pathology. In the present study, we sought to confirm the elevation of CSF sPDGFRβ and association with BBB leakiness in AD and to assess the relationship to markers of disease pathology, i.e. reduced Aβ42 and elevated total tau (t-tau) and phosphorylated tau (p-tau).

## Methods

### Study cohort

CSF sPDGFRβ and albumin were measured in 39 AD cases and 39 controls for which CSF t-tau, p-tau_181P_ and Aβ42 (Cat no 81583, 81551 and 81576 respectively) had previously been determined using commercially available sandwich enzyme-linked immunosorbent assays (ELISAs) (INNOTEST, Fujirebio, Ghent, Belgium). All AD patients had abnormal CSF levels of the core AD biomarkers (t-tau > 400 pg/mL, p-tau > 60 pg/mL and Aβ42 < 550 pg/mL), while controls had normal levels (t-tau = 234.67 ± SD 11.42; p-tau 40.28 ± 1.58; Aβ42 834.18 ± 32.67). The cut-off values used in this study are in line with current clinical practice and closely resemble those outlined by Hansson et al. [9]. The mean ages were 76.21 years in the AD cases (SD 6.11) and 68.21 years in the controls (SD 11.96). The proportions of males and females were similar in the AD (25M, 14F) and control (23M, 16F) cohorts. The demographic characteristics for each cohort are summarised in Table 1. The present assays were performed on de-identified left-over aliquots from clinical diagnostic CSF samples and followed the Swedish Biobank law (Biobanks in Medical Care Act) and procedures approved by the Ethical Committee at University of Gothenburg. Cognitive data and *APOE* status were not recorded, and matching serum samples were not available for this cohort.

**Table 1:** Summary demographics of the AD/control cohort

	*n*	Gender (M:F)	Age (mean years ± SD)	Aβ42 (mean ng/L ± SEM)	p-tau (mean ng/L ± SEM)	t-tau (mean ng/L ± SEM)
Control	39	23:16	68.2 ± 11.9	834.18 ± 32.67	40.28 ± 1.58	234.67 ± 11.42
AD	39	25:14	76.2 ± 6.11	435.18 ± 11.88	84.79 ± 3.21	725.69 ± 36.31

sPDGFRβ was, however, also measured in an independent CSF and matched serum sample cohort from neurologically normal controls (*n* = 23) spanning a larger age range (23–84 years). The CSF/serum albumin ratio had previously been determined by an immunoturbidimetric albumin method on Elecsys (Roche Diagnostics, Penzberg, Germany). The demographics of this cohort are presented in Table 2.

**Table 2:** Summary demographics of the independent neurologically normal CSF/serum matched cohort

	*n*	Gender (M:F)	Age (mean years ± SD)	Albumin ratio
CSF/serum cohort	23	12:11	58.13 ± 20.25	9.04 ± 1.19

### sPDGFRβ measurement in CSF and serum by sandwich ELISA

CSF and serum sPDGFRβ levels were measured by sandwich ELISA (Invitrogen Cat no EHPDGFRB, Thermo Fisher Scientific, Loughborough, UK). CSF samples (100 μL undiluted) and serum (diluted 1 in 40 in proprietary dilution buffer supplied with the kit) were loaded. Standards, samples and blanks were added in duplicate. Absorbance was read at 450 nM in a FLUOstar OPTIMA plate reader (BMG labtech, Aylesbury, UK). Reproducibility reported in the datasheet indicates an inter-assay CV < 12% and intra-assay < 10%, with spike recovery between 90 and 110% for serum, plasma and cell culture media. PDGFRβ concentration in samples was calculated by interpolation against the standard curve for each case, derived from serial dilutions of recombinant PDGFRβ (18,000–24 pg/mL). The mean values from duplicate measurements are presented.

### Albumin measurement in CSF by sandwich ELISA

CSF albumin level was measured by sandwich ELISA (Cat no 108788) (Abcam, Cambridge, UK). CSF samples were diluted 1 in 2000 in proprietary dilution buffer supplied with the kit. Standards, samples and blanks were added in duplicate. Absorbance was read at 450 nM in a FLUOstar OPTIMA plate reader (BMG labtech, Aylesbury, UK). Albumin concentration in each sample was interpolated from a standard curve derived from serial dilution of recombinant human albumin (200–3.125 ng/mL). The mean values are presented.

### Statistical analysis

As the CSF sPDGFRβ and albumin levels were normally distributed, unpaired two-tailed *t* tests or ANOVA with Bonferroni post hoc correction was used for comparisons between groups, and Pearson’s test was used to assess linear correlation. Data values outside the 99% confidence interval of the linear regression line were considered to be outliers and removed prior to statistical analysis. The analyses used SPSS version 16 (SPSS, Chicago) and GraphPad Prism version 6 (GraphPad Software, La Jolla, CA). *p* values < 0.05 were considered statistically significant.

## Results

**Fig. 1:**
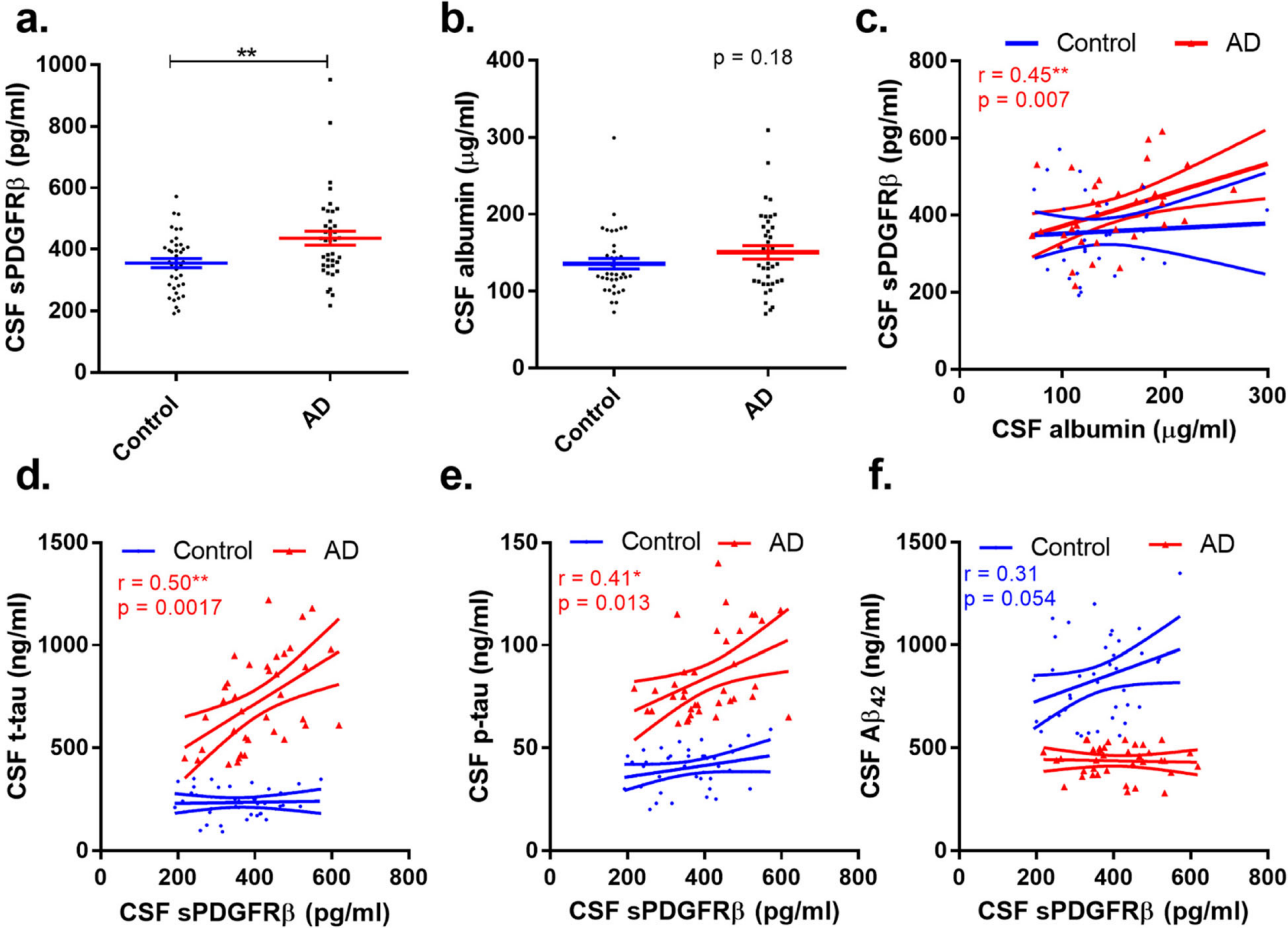
CSF soluble PDGFRβ level is increased in Alzheimer’s disease (AD) in relation to CSF markers of BBB leakiness (CSF albumin) and disease pathology (elevated total tau (t-tau) and phosphorylated tau (p-tau)). **a** Dot plot showing significantly higher levels of CSF sPDGFRβ in AD compared to controls (*n* = 39 per group) (*p* = 0.0038). **b** Dot plot showing CSF albumin level in the AD and controls (*p* = 0.18). **c** Scatterplots showing positive correlation between CSF sPDGFRβ and CSF albumin in AD. **d**–**f** Scatterplots showing a positive correlation between CSF sPDGFRβ and t-tau and p-tau but not with Aβ_42_ in AD. In **a** and **b**, the bars represent the mean ± SEM. In **c**–**f**, the best-fit linear regression line is shown and 95% confidence intervals are superimposed. Each dot represents an individual sample. *p* < 0.05 was considered statistically significant

CSF sPDGFRβ levels were significantly higher in AD than in controls (mean 426.7 pg/mL ± SD 20.9 in AD v 355.6 pg/mL ± 14.9 in controls) (*p* = 0.0038) (Fig. 1a). Mean CSF albumin level was higher in AD than in controls, but the difference did not reach statistical significance (150.1 μg/mL ± 8.6 in AD v 135.6 μg/mL ± 6.9 in controls) (Fig. 1b).

CSF sPDGFRβ correlated with albumin in the AD cohort (*r* = 0.45, *p* = 0.007) but not in controls (*r* = 0.059, *p* = 0.74) (Fig. 1c). Inspection revealed two obvious outliers that were both well outside the 99% confidence interval of the linear regression line and were excluded from analysis.

CSF sPDGFRβ correlated positively with t-tau in AD (*r* = 0.50, *p* = 0.0017) but not in controls (*r* = 0.03, *p* = 0.83) (Fig. 1d), and with p-tau in AD (*r* = 0.41, *p* = 0.013) but not in controls (*r* = 0.26, *p* = 0.10) (Fig. 1e). CSF sPDGFRβ did not correlate with the CSF Aβ42 level in the control cohort (*r* = 0.31, *p* = 0.054) or AD cohort (*r* = − 0.04, *p* = 0.80) (Fig. 1f).

The control and AD groups did not differ significantly according to age, and sPDGFRβ did not vary with age within the controls (*r* = 0.10, *p* = 0.54) or AD (*r* = 0.29, *p* = 0.077) groups (Additional file 1: Figure S1a). CSF sPDGFRβ did not differ between gender in the controls or AD cohort (Additional file 1: Figure S1b).

**Fig. 2:**
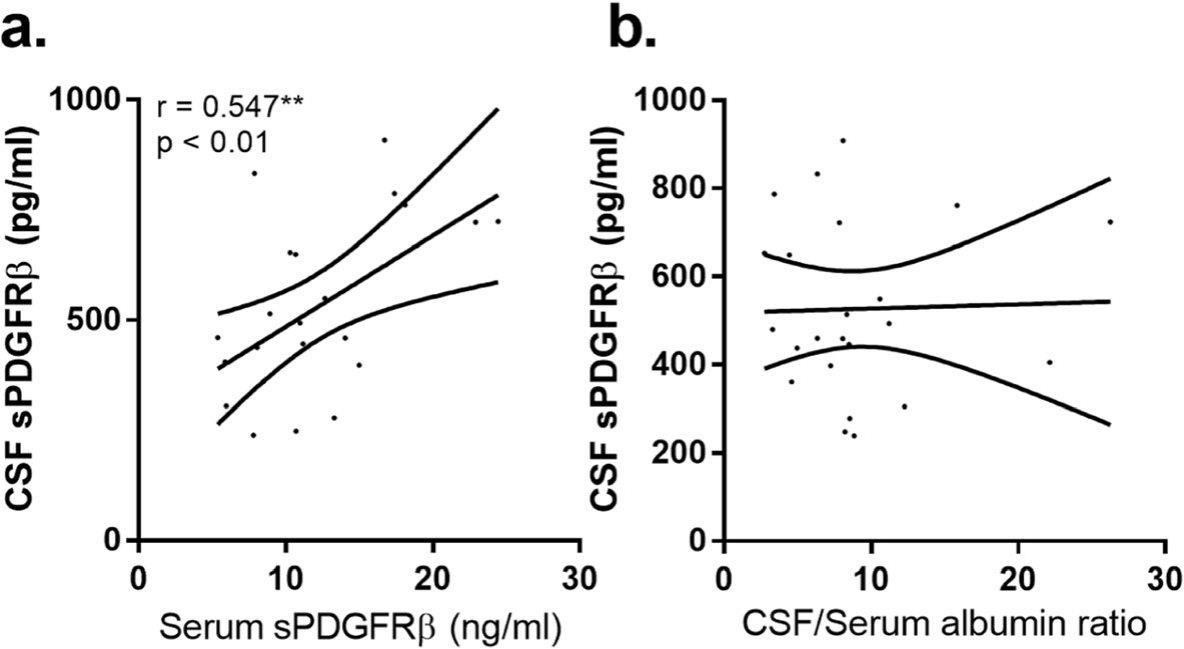
Relationship between CSF and serum sPDGFRβ and the CSF/serum albumin ratio in an independent neurologically normal cohort (*n* = 23) of matched CSF and serum. **a** Scatterplot showing a positive correlation between serum and CSF sPDGFRβ. **b** Scatterplot showing no relationship between CSF sPDGFRβ and the CSF/serum albumin ratio. The best-fit linear regression line is shown and 95% confidence intervals are superimposed. Each dot represents an individual sample. *p* < 0.05 was considered statistically significant

We also measured sPDGFRβ in a separate non-AD cohort of matched CSF and serum sample from neurologically heathy individuals. CSF and serum sPDGFRβ level were positively correlated (*r* = 0.547, *p* = 0.0085) (Fig. 2a). CSF sPDGFRβ did not correlate with the CSF/serum albumin ratio (Fig. 2b).

## Discussion

We have confirmed that CSF sPDGFRβ is increased in AD, to an extent that correlates with CSF albumin level (a marker of BBB leakiness) and with the neurodegeneration biomarkers CSF t-tau and p-tau. We have also shown that the level of sPDGFRβ in CSF correlates with that in serum in an independent cohort of matched CSF/serum samples. Our findings support recent reports of pericyte injury and BBB leakiness in AD, as demonstrated in histological and biochemical studies of autopsy tissue [4] and in biomarker and imaging studies in AD patients [7]. Nation et al. recently reported that elevated CSF sPDGFRβ was associated with cognitive decline independent of the levels of Aβ42 and tau in the early stages of AD [6]. Here we have shown that in an AD cohort, elevated sPDGFRβ correlated with albumin, a CSF marker of BBB leakiness, and with t-tau and p-tau levels but not with Aβ42. Our novel finding of a correlation between sPDGFRβ levels in the serum and CSF raises the possibility of using serum sPDGFRβ measurements to track the progression of pericyte injury and BBB breakdown in AD.

Post-mortem studies have confirmed pericyte loss and BBB leakiness in AD [3, 10]. Reduction in PDGFRβ is associated with biochemical evidence of cerebral hypoperfusion and BBB breakdown and with Aβ42 level in post-mortem tissue [4]. The reduction in PDGFRβ is most pronounced in brains from APOE ε4-positive AD patients [3, 4]. Pdgfrβ^F7/F7^ mice, which have disrupted PDGFRβ signalling, have a more pronounced vascular phenotype associated with vascular loss and BBB leakiness [11], and when Pdgdfβ^+/−^ heterozygous mice were crossed with Tg-APP mice, there was accelerated Aβ accumulation and clinical disease progression [8], supporting recent studies indicating that vascular dysfunction is one of the earliest pathological features in pre-clinical AD [12].

Compromised BBB integrity that can be demonstrated in the hippocampus in normal ageing and is exacerbated in MCI, as revealed by advanced dynamic contrast-enhanced MRI, is associated with elevated CSF sPDGFRβ [7]. Nation et al. reported that elevated CSF sPDGFRβ in mild cognitive impairment was related to BBB integrity but was independent of CSF Aβ42 or tau [6]. Elevated CSF sPDGFRβ likely reflects release of a soluble fragment of pericyte PDGFRβ, due to its cleavage by ADAM-10, as was shown in vitro in response to simulated ischemia or Aβ peptide [5, 6]. Here we show that elevation of CSF sPDGFRβ in AD is associated with BBB leakiness but also correlates with established CSF markers of AD progression, i.e. elevated CSF t-tau and p-tau, although as in the Nation et al. study we did not detect a direct relationship between CSF sPDGFRβ and Aβ42. Larger cohort studies are needed to assess the value of elevated sPDGFRβ in CSF, and perhaps even serum, as a marker of progressive cognitive dysfunction and the development of AD.

The mechanisms of pericyte injury in AD remain unclear, but both Aβ and hypoperfusion are probably contributors. Aβ peptides at supraphysiological concentrations were shown to be toxic to human brain pericytes [13], dependent on *APOE* genotype [14]. The toxic effects are probably related to the conformational assembly and species of Aβ [15]. In a bilateral carotid artery stenosis (BCAS) model that mimics chronic cerebral hypoperfusion, pericyte loss and BBB dysfunction in the corpus callosum preceded white matter injury and cognitive decline [16]. The precise regional distribution and timing of pericyte injury in AD, particularly in relation to other pathological manifestations of the disease, has still to be determined.

## Conclusions

This study has several limitations including the small number of AD cases and controls and the paucity of clinical details relating to the de-identified AD cases, such as dementia severity, disease duration and *APOE* genotype, that would be of interest for further analysis. This is also an observational cross-sectional study and does not provide clues as to the timing and regional changes in pericyte loss in relation to onset and progression of disease. The extent of vascular burden within our cohort is also unclear, and as in other clinical studies, it is likely to include a spectrum of AD and mixed dementia cases. Other variables that related to the collection of the samples and might theoretically impact on the measurements (e.g. time of day, needle type) were also not available for analysis. Nonetheless, our data together with recent studies suggest that markers of vascular dysfunction, including pericyte loss and BBB leakiness, are related to cognitive impairment in MCI and disease pathology in AD and can potentially be monitored by analysis of CSF and possibly also serum. The inclusion of vascular biomarkers as part of an AD research framework, as suggested by Sweeney and colleagues [17], would improve our understanding of AD pathophysiology and may prove useful to identify those AD patients for whom tailored therapies to reduce vascular burden may offer a more effective treatment.

## Additional file

Additional file 1: **Figure S1** CSF-sPDGFRβ is not altered in relation to age or gender. (a) Scatterplot showing no statistically significant relationship between CSF-sPDGFRβ level and age in AD (red) and control (green). The best-fit linear regression line and 95% confidence interval for each group are superimposed. (b) Bar chart showing CSF-sPDGFRβ level in control and AD group stratified for gender. No significant differences were observed. Bars represent the mean ± SEM. (DOCX 209 kb)

## Data Availability

All data generated or analysed during this study are included in this published article [and its supplementary information files].

## Abbreviations

AD: Alzheimer’s disease
Aβ42: Amyloid-beta 1–42
BBB: Blood-brain barrier
CSF: Cerebrospinal fluid
PDGFRβ: Platelet-derived growth factor receptor beta
p-tau: Phosphorylated tau
sPDGFRβ: Soluble platelet-derived growth factor receptor beta
t-tau: Total tau
