# Supplementary material for: CSF evidence of pericyte damage in Alzheimer’s disease is associated with markers of blood-brain barrier dysfunction and disease pathology
Source: Alzheimers Res Ther. 2019 Sep 14;11:81. doi: 10.1186/s13195-019-0534-8 (PMC6745071; doi:10.1186/s13195-019-0534-8)
Supplement: Supplementary file 1 — Figure S1 CSF-sPDGFRβ is not altered in relation to age or gender. (a) Scatterplot showing no statistically significant relationship between CSF-sPDGFRβ level and age in AD (red) and control (green). The best-fit linear regression line and 95% confidence interval for each group are superimposed. (b) Bar chart showing CSF-sPDGFRβ level in control and AD group stratified for gender. No significant differences were observed. Bars represent the mean ± SEM. (DOCX 209 kb) [file 13195_2019_534_MOESM1_ESM.docx]

**
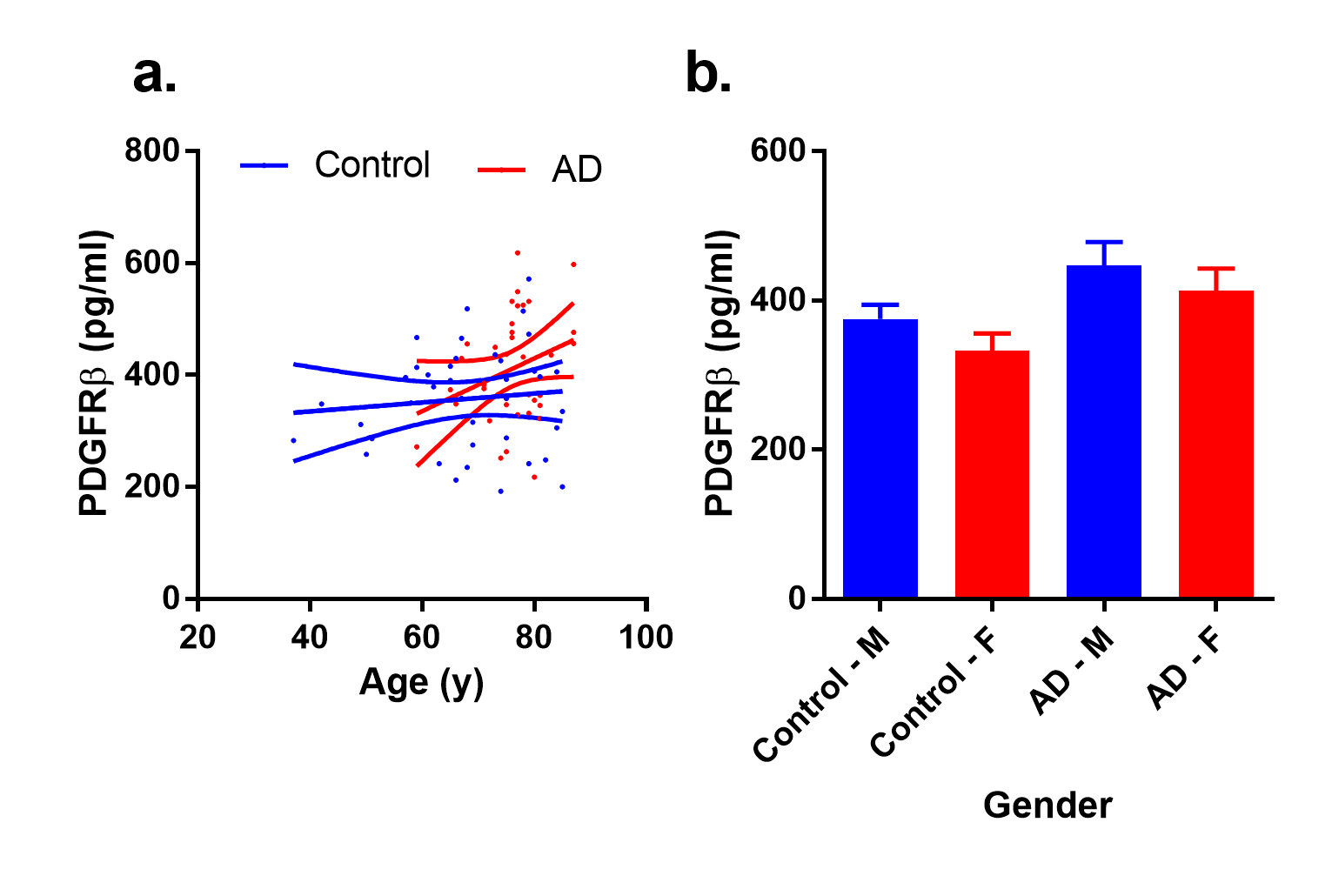
**

Additional file 1: **Figure S1** CSF-sPDGFRβ is not altered in relation to age or gender. (a) Scatterplot showing no statistically significant relationship between CSF-sPDGFRβ level and age in AD (red) and control (green). The best-fit linear regression line and 95% confidence interval for each group are superimposed. (b) Bar chart showing CSF-sPDGFRβ level in control and AD group stratified for gender. No significant differences were observed. Bars represent the mean ± SEM.
